# Drug–Drug Interactions in Italian Patients with Chronic Hepatitis C Treated with Pangenotypic Direct Acting Agents: Insights from a Real-World Study

**DOI:** 10.3390/ijerph18137144

**Published:** 2021-07-03

**Authors:** Alessandra Mangia, Francesco Scaglione, Pierluigi Toniutto, Mario Pirisi, Nicola Coppola, Giovanni Di Perri, Gema Alvarez Nieto, Stefano Calabrese, Candido Hernandez, Valentina Perrone, Luca Degli Esposti, Stefano Fagiuoli

**Affiliations:** 1 Liver Unit, Fondazione “Casa Sollievo Della Sofferenza” IRCCS, 71013 San Giovanni Rotondo, Italy; a.mangia@tin.it; 2 Department of Oncology and Onco-Hematology, University of Milan, 20122 Milan, Italy; francesco.scaglione@unimi.it; 3 Hepatology and Liver Transplantation Unit, Azienda Ospedaliero Universitaria, 33100 Udine, Italy; pierluigi.toniutto@uniud.it; 4 Department of Translational Medicine (DiMeT), Università del Piemonte Orientale, 28100 Novara, Italy; mario.pirisi@med.unipmn.it; 5 Department of Mental Health and Public Medicine–Infectious Diseases Unit, University of Campania Luigi Vanvitelli, 81100 Caserta, Italy; nicola.coppola@unicampania.it; 6 Department of Medical Sciences, University of Turin, 10124 Turin, Italy; giovanni.diperri@unito.it; 7 Gilead Sciences, Medical Affairs Italy, 202124 Milan, Italy; gema.alvareznieto@gilead.com (G.A.N.); stefano.calabrese@gilead.com (S.C.); 8 Gilead Sciences, Global Medical Affairs, Stockley Park, London UB11 1BD, UK; Candido.Hernandez@gilead.com; 9 CliCon S.r.l. Health, Economics & Outcomes Research, 40137 Bologna, Italy; luca.degliesposti@clicon.it; 10 Gastroenterology and Transplant Hepatology, Papa Giovanni XXIII Hospital, 24127 Bergamo, Italy; sfagiuoli@asst-pg23.it

**Keywords:** drug–drug interactions, glecaprevir/pibrentasvir, HCV, sofosbuvir/velpatasvir

## Abstract

This Italian observational real-world study aims to assess in chronic hepatitis C virus (HCV) patients treated with pangenotypic direct acting agents (pDAAs) glecaprevir/pibrentasvir (GLE/PIB) or sofosbuvir/velpatasvir (SOF/VEL) the potential drug–drug interactions (DDIs) with concomitant medications prescribed, with a focus on cardiovascular and system nervous (CNS) co-medications. Data were collected from administrative databases covering 6.9 million health-assisted individuals. All patients prescribed SOF/VEL or GLE/PIB between 11/2017 and 12/2018 were included. Patients were analyzed while on DAA. DDIs were identified according to the Liverpool University tool. Overall, 3181 HCV patients were included: 1619 in the GLE/PIB cohort and 1562 in the SOF/VEL cohort. SOF/VEL patients were generally older than GLE/PIB ones (mean age 58.4 vs. 53.1, *p* < 0.001) and had more cardiovascular and CNS comorbidities (58% vs. 42%, *p* < 0.001 and 33% vs. 28%, *p* = 0.002, respectively). Contraindications due to DDIs in the GLE/PIB cohort affected 9.3% and 3.2% of patients before and on DAA, respectively, while the percentages in the SOF/VEL cohort were 3.2% before and 0.4% after pDAAs initiation. Among GLE/PIB patients, 2.7% had cardiovascular drugs (all statins) contraindicated while on DAA. The potential DDIs between cardiovascular drugs and SOF/VEL were mainly with statins (5%). SOF/VEL was prescribed in patients with older age and with more cardiovascular and CNS comorbidities. Despite this, a proportion of contraindicated drugs lower than that of GLE/PIB was registered.

## 1. Introduction

Chronic hepatitis C virus (HCV) infection is a global medical and public health concern, with approximately 71 million people estimated to live with HCV worldwide [1]. HCV is one of the main causes of chronic liver disease, cirrhosis, hepatocellular carcinoma, and liver transplant [2]. Chronic HCV infections may remain asymptomatic for decades, and symptoms can occur at a late stage [3]. 

In the last few decades, the pharmacological armamentarium for treating HCV infection has improved dramatically with the introduction of oral direct-acting antivirals (DAAs), which demonstrated both high efficacy and high tolerability in clinical trials and in real clinical practice [4]. The advent of a new generation of pangenotypic oral DAAs (pDAAs) marked a second breakthrough, with a therapeutic combination regimen of more agents administered as fixed combination [5]. Currently available pDAA regimens are able to achieve a sustained virological response (SVR) in more than 98% of patients regardless of previously recognized negative predictors of positive treatment response [6]. In Italy, the DAAs currently reimbursed for all HCV patients from 2017 are elbasvir/grazoprevir (from February 2017), sofosbuvir/velpatasvir (SOF/VEL) (from May 2017), and glecaprevir/pibrentasvir (GLE/PIB) (from October 2017) [4].

pDAAs carry the drawback of potential drug–drug interaction (DDI) with concomitant medication, which can significantly alter the drug’s exposure and lead to serious clinical implications due to a decreased drug efficacy or an increased drug toxicity [7]. The potential of a DDI is of special consideration, since pDAAs treatment regimens contain up to five drugs, each one potentially acting as substrates, inhibitors, and/or inducers of metabolic enzymes and transporters [8,9]. According to the recently updated European Association for the Study of the Liver (EASL) recommendations on the treatment of HCV [10], a thorough DDI risk assessment is required for patients before starting treatment with pDAAs and before starting other co-medications during treatment exposure. Furthermore, since pDAAs are recommended to the entire HCV population, the diversity of HCV patients eligible to such treatments is expected to increase over the years in terms of age, co–morbidity profile, and polypharmacy regimens [6]. Clinical trials that investigated the DDI between DAAs and some key drugs generally included patients with limited concomitant medications [11,12]; however, in real life clinical practice, the presence of polypharmacology regimens can make the HCV treatment more challenging than expected [13,14,15]. Indeed, studies performed in real-world settings observed among HCV patients high rates of comorbidities and the use of concomitant medication, especially in older ones, which means a greater exposure to potential DDI; moreover, the most commonly concomitant drugs were reported to belong to cardiovascular and central nervous system classes [6,15,16,17].

Studies based on real-world data could better help to understand how the potential DDIs are managed in clinical practice outside the controlled settings of clinical trials and to evaluate the therapeutic appropriateness of the DAAs therapy prescribed, based on prescribing information for each therapy, their key interactions, and the presence of concomitant medication.

The aims of the present real-world study are to determine among HCV patients in therapy with SOF/VEL or GLE/PIB treatments the potential DDIs between pDAAs and other concomitant treatments administered, and to evaluate the modification of the pharmaco–utilization of these co-treatments during pDAAs exposure, with particular reference to cardiovascular and nervous system drug classes. Moreover, we evaluated if different demographic and clinical characteristics exist between patients prescribed SOF/VEL and those with GLE/PIB. 

## 2. Materials and Methods

### 2.1. Data Sources

This observational retrospective cohort study was performed by integrating the administrative and laboratory data flows of a dataset of individuals with at least a CV risk factor (detected from 2010 to 2018) from a pool of Italian Healthcare Entities geographically distributed throughout the national territory, covering a total of 6.9 million health-assisted individuals (approximately 11.4% of the Italian population). In a feasibility analysis based on a sample of Italian Healthcare Entities, around 99% of patients with at least a prescription for SOF/VEL or GLE/PIB had at least a cardiovascular record. Therefore, the included patients were representative of the overall sample analyzed. To perform the analysis, the following databases have been used: a demographic database that contains patients’ demographic data; a pharmaceuticals database providing data on prescription as ATC (Anatomical–Therapeutic Chemical) code, number of packages, number of units per package, unit cost per package, and prescription date; hospitalization database that includes all hospitalization data with discharge diagnosis codes classified according to the International Classification of Diseases, Ninth Revision, Clinical Modification (ICD–9–CM), Diagnosis Related Group (DRG), and DRG-related charge (provided by the Health System); the outpatient specialist services database, which contains date of prescription, description activity of diagnostic tests, and visits for patients in analysis and laboratory tests or specialist visit charges; the exemption ticket for the pathology database that includes disease exemption codes and the dates of exemption.

The patient code in each database allowed electronic linkage between all different databases. To guarantee patients’ privacy, an anonymous univocal numeric code was assigned to each subject included in the study, in full compliance with the European General Data Protection Regulation (GDPR) (2016/679). No identifiers related to patients were provided to the authors. All the results of the analyses were produced as aggregated summaries, which are not possible to assign, either directly or indirectly, to individual patients. Informed consent was not required, since obtaining it is impossible for organizational reasons (pronouncement of the Data Privacy Guarantor Authority, General Authorization for personal data treatment for scientific research purposes—n.9/2014). According to the Italian law, this study has been notified and approved by each competent Ethics Committees of each Healthcare Entity involved in the study (as reported in the Institutional Review Board Statement, below).

### 2.2. Cohorts Definition and Study Variables

All individuals that were prescribed SOF/VEL (ATC code: J05AP55) or GLE/PIB (ATC code: J05AP57) between November 2017 and December 2018 were included in the study. The first pDAAs prescription within the inclusion period was indicated as the index date. The year before index date was defined as the pre-DAA period, while the follow-up period corresponded to the duration of pDAAs treatments started at the index date (on DAA). Specifically, the duration of pDAAs treatments was calculated based on the number of packages dispensed considering the days covered by each prescription. Based on the pDAAs prescribed, two cohorts were created: the SOF/VEL and the GLE/PIB cohorts.

The time since diagnosis was evaluated considering the entire available period before the index date as time since first anti-HCV antibodies or HCV viral load or genotyping or HCV diagnosis, whichever occurred first. HCV diagnosis was identified by discharge diagnosis for HCV (ICD–9–CM codes: 070.4, 070.5, 070.7) or by the presence of an exemption code or HCV drugs prescription (ATC code: J05AP).

The presence of comorbidities was evaluated in the pre-DAA period. Patients have been characterized based on the Charlson comorbidity index, which assigns a score to each concomitant disease based on drugs treatment and hospitalizations; therefore, untreated/non-hospitalized comorbidities were not captured. Cardiovascular comorbidities were identified by the use of ATC B (blood), C (cardiovascular system), A10 (diabetes), or hospitalizations for major diagnostic categories (MDC) 5 (cardiovascular system), while central nervous system comorbidities were ascertained by the use of ATC N or hospitalizations for MDC 1. In addition, the presence of cirrhosis (identified by ICD–9–CM code: 571 or by using exemption code 008), hepatocellular carcinoma (ICD–9–CM code: 155), and liver transplant (procedure code 505) were evaluated during the characterization period.

Co-treatments were investigated in the pre-DAA period as well as while on DAA by the presence of prescriptions for co-treatments dispensed in the respective time windows. All possible co-treatments were classified based on the first-level ATC code. The analyses focused on the concomitant cardiovascular therapies as antihypertensives (ATC code: C02), diuretics (ATC code: C03), beta-blocking agents (ATC code: C07), calcium channel blockers (ATC code: C08), agents acting on the renin-angiotensin system (ATC code: C09)], lipid-lowering drugs (ATC code: C10), antiplatelets/anticoagulants (ATC code: B01), hypoglycemic agents (ATC code: A10), and nervous system therapies (ATC code: N).

The severity levels of the potential interacting drugs were identified according to the Liverpool University tool [18], which is an international resource also recommended by EASL [10]. All prescribed co-treatments were initially identified and, in accordance with Liverpool University indications, were classified into four categories based on the severity of each interaction as follows:

1. no interaction expected;
2. potential weak interaction (for drugs in this category, additional action could not be required);
3. potential interaction (for drugs in this category, dose adjustment or additional monitoring may be required);
4. contraindicated (these drugs are contraindicated and/or should not be co-administered).

If a patient had multiple co-treatments with different interaction profiles, the most severe interaction was considered.

All drugs with potential weak interactions, potential interactions, and contraindicated were identified as both pre-DAA and on-DAA periods.

The pharmacoutilization of cardiovascular and nervous system co-treatments was determined before and after the index date in terms of persistence, adherence, and treatment changes. Specifically, persistence was defined as the presence of the same drug prescription during pre-DAA and on-DAA periods; adherence was defined as therapeutic coverage for ≥80% of days during pDAA exposure; to evaluate the dose decrease, at first, the daily doses were calculated as overall milligrams dispensed divided by the number of therapeutic coverage days of prescriptions during the pre-DAA and on-DAA period, respectively. Then, the daily dose pre-DAA and on-DAA were compared to assess if a dose decrease was present; discontinuation was considered as either a switch of therapy (the presence of another drug of the same class administered pre-DAA during DAA exposure) or as interruption of a drug class prescribed pre-DAA during follow-up; resumption was observed in patients using the same drug pre-DAA and after DAA exposure that interrupted it while on-DAA.

### 2.3. Statistical Analysis

Continuous variables were reported as mean ± standard deviation (SD); categorical variables were expressed as numbers and percentages. Clinical and demographic characteristics were evaluated and compared between SOF/VEL and GLE/PIB patients. The T-student test was used to compare continuous variables and the chi square test was used for categorical ones. Statistical significance was accepted at *p* < 0.05. All analyses have been performed using STATA SE version 12.0.

## 3. Results

A total of 1562 SOF/VEL patients and 1619 GLE/PIB patients were included in the respective cohorts (male 58.3% and 59.5% respectively *p* = 0.530). SOF/VEL patients were older than GLE/PIB (mean age 58.4 ± 15.8 vs 53.1 ± 15.6, respectively *p* < 0.001). Characteristics of the cohorts at baseline are presented in Table 1. The SOF/VEL cohort had a more serious comorbidity profile than the GLE/PIB cohort: The Charlson Index of SOF/VEL patients was 0.9 ±1.3, while the one of the GLE/PIB cohort was 0.6 ± 1.0 (*p* < 0.001). Moreover, cardiovascular and nervous system comorbidities were more frequent among SOF/VEL than GLE/PIB patients: 58.2% vs. 42.3% (*p* < 0.001) and 33.4% vs. 28.2% (*p* = 0.002), respectively. In the SOF/VEL cohort, the proportion of patients with cirrhosis was 15.2% and that with hepatocellular carcinoma was 1.9%, which is higher in comparison with the GLE/PIB cohort, in which 7.2% of patients had cirrhosis (*p* < 0.001) and 0.3% had hepatocellular carcinoma (*p* < 0.001). No liver transplant patient was observed at baseline among GLE/PIB patients.

Time since HCV diagnosis was similar in both cohorts: median (IQR) years of diagnosis was 3.3 (6.2) and 3.4 (6.1) for SOF/VEL and GLE/PIB patients, respectively. Around 37.5% of SOF/VEL and 38.8% of GLE/PIB users have been diagnosed for over 5 years, while 28.8% (SOF/VEL) and 29.0% (GLE/PIB) have been diagnosed between 1 and 5 years. The mean time of follow-up was 12 weeks for SOF/VEL and 8.4 weeks for GLE/PIB patients. Specifically, pDAAs exposure was 12 weeks for SOF/VEL (99.7%) and 8 weeks (90.2%), 12 weeks (8.9%), and 16 weeks (0.9%) for GLE/PIB patients.

**Figure 1:**
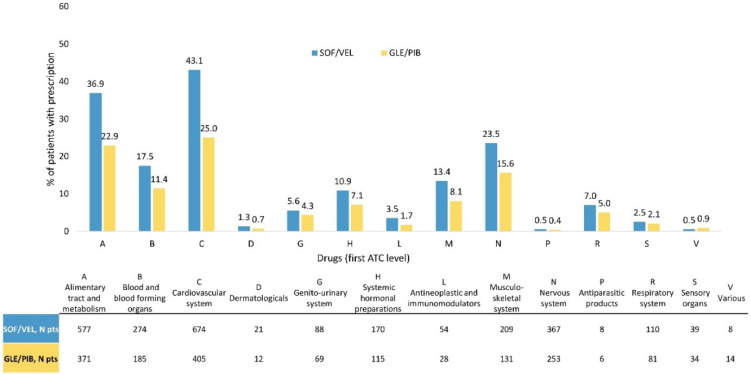
Co-treatment (classified as first-level ATC code) prescribed while on DAA in SOF/VEL and GLE/PIB cohorts. Abbreviation: SOF/VEL: sofosbuvir/velpatasvir; GLE/PIB: glecaprevir/pibrentasvir.

During the pDAAs exposure, the co-treatments most frequently observed among all included patients belonged to the class of cardiovascular system (33.9%), followed by alimentary tract and metabolism (29.8%) and nervous system (19.5%). These three classes were more frequently prescribed to SOF/VEL than GLE/PIB patients: 43.1% vs. 25% for cardiovascular system, 36.9% vs. 22.9% for alimentary tract and metabolism, and 23.5% vs. 15.6% for nervous system therapeutic classes, respectively (Figure 1).

**Figure 2:**
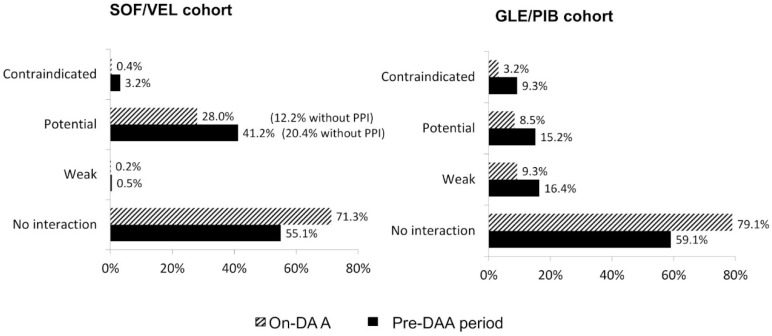
Severity of DDIs classified according to the Liverpool University tool reported in the pre-DAA and on-DAA period in SOF/VEL (left panel) and GLE/PIB (right panel) cohort. Abbreviation: GLE/PIB: glecaprevir/pibrentasvir; PPI, proton pump inhibitors; SOF/VEL: sofosbuvir/velpatasvir.

As shown in Figure 2, contraindications due to DDIs remain higher in GLE/PIB cohorts both before and during DAA treatment (9.3% and 3.2%, respectively), while patients with contraindicated co-treatments decreased from 3.2% to 0.4% after pDAAs initiation in the SOF/VEL cohort. Potential DDIs in SOF/VEL users decreased from 41.2% to 28.0% (15.8% were related to proton pump inhibitors (PPIs) prescribed while on-DAA), potential weak DDIs were 0.5% before index date and 0.2% during follow-up. The proportion of GLE/PIB patients with potential DDI reduced from 15.2% to 8.5%, with potential weak DDIs from 16.4% to 9.3%. In both cohorts, the percentage of co-treated patients with no interaction expected increased from 55.1% to 71.3% (SOF/VEL) and from 59.1% to 79.1% (GLE/PIB).

**Figure 3:**
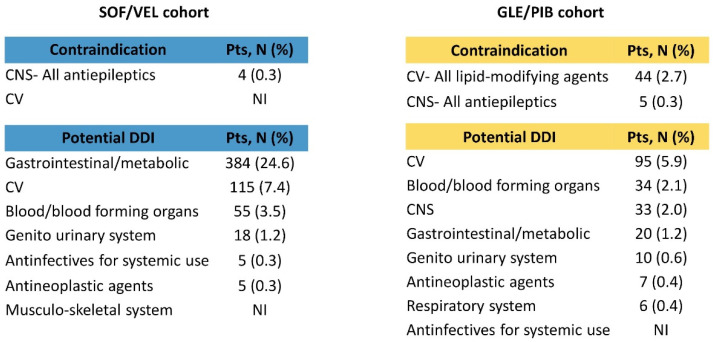
Co-medications contraindicated or with potential DDIs administered to patients during pDAAs exposure by DDIs in SOF/VEL (left panel) and GLE/PIB (right panel) cohorts. Abbreviations: CNS, central nervous system; CV, cardiovascular DDI, drug–drug interaction; GLE/PIB, glecaprevir/pibrentasvir; PPI, proton pump inhibitors; Pts, patients; SOF/VEL, sofosbuvir/velpatasvir; NI, not issuable for data privacy (less than four patients).

Co-medications contraindicated or with potential clinically significant DDIs with a focus on cardiovascular and nervous system classes are reported in Figure 3 for both cohorts. A low number of contraindications with concomitant nervous system drugs were found for both pDAAs and were all related to antiepileptics. For cardiovascular drugs, in the GLE/PIB cohort, a remarkable number of contraindications (2.7%) were found, all of them with lipid-modifying agents. Potential DDIs for cardiovascular concomitant drugs in the SOF/VEL population were mainly due to lipid-modifying agents (5% of the overall cohort). For GLE/PIB, the cardiovascular drugs with potential DDIs (5.9%) were related to blood pressure medications (renin–angiotensin–aldosterone system inhibitors, beta–blocking agents, and calcium–channel blockers, all accounting for approximately 4% of the total overall cohort) while nervous system drugs with potential DDIs (2%) were mainly antipsychotics (1.3%), with quetiapine involved in 1.1% of cases, and analgesics (0.7%, of which 0.6% represented by oxycodone). The number of patients treated with the most common concomitant drugs belonging to nervous system (quetiapine/oxycodone) and cardiovascular (atorvastatin/simvastatin) classes are reported in Supplementary Figure S1.

In the SOF/VEL cohort, all patients who before initiating the pDDAs treatment received contraindicated cardiovascular treatments discontinued such treatments; similarly, the majority of patients (14 over 17) with nervous system agents with contraindication did not receive them during SOF/VEL exposure (Table 2). A different trend was found in the GLE/PIB cohort, in which a non-negligible proportion of patients with contraindicated cardiovascular (N = 42 over 136 patients) and nervous system (N = 4 over 15 patients) co-treatments were persistent with them after initiating the pDAA and were adherent to these medications while on treatment, although a dose decrease was observed in almost all patients. Of the 94 patients discontinuing the contraindicated CV drug, 41 resumed such therapies after GLE/PIB exposure. Patients with contraindicated cardiovascular treatments mainly received lipid-modifying drugs: 40% of simvastatin and 25.8% atorvastatin users kept being treated with such drugs after starting GLE/PIB, almost all of them at a decreased dosage. Moreover, 10 out of the 24 patients that discontinued simvastatin and 29 out of 63 patients that discontinued atorvastatin re-started these therapies after DAAs exposure.

## 4. Discussion

The new DAAs, although safe and effective, present an important number of drug interactions, which if not properly evaluated could compromise the outcome of the treatment. In the present real-world study, we aimed to investigate the management of HCV patients prescribed pDAAs in clinical practice in Italian settings. Specifically, we found that in our study population, there were differences between patients receiving a first SOF/VEL or GLE/PIB prescription, especially in terms of age, comorbidity profile, previous liver complications, and co-treatments. Moreover, we explored how the co-treatments were managed in clinical practice to avoid serious DDIs with HCV therapies.

Characteristics at baseline in both cohorts revealed the presence of comorbidities and mean age over 50 years old, which increases the risk of DDIs when receiving HCV treatment. Our data are consistent with real-world HCV patients treated with pDAAs described in the literature [15]. Moreover, such characteristics collected in routine clinical practice are far from the stringent inclusion criteria of clinical trials and provide a more real profile of HCV patients initiating DAA therapies. The SOF/VEL cohort have a worse clinical profile as patients are older, with more comorbidities and with more concomitant treatments than the GLE/PIB cohort, thus suggesting SOF/VEL is more likely prescribed in multi-treated patients with comorbidities.

The concomitant treatments prescribed were in line with the results reported in a Spanish real-world study, in which the most consumed therapeutic groups belonged to the class of alimentary tract and metabolism (37.5%), cardiovascular system (37.5%), and nervous system (34.1%) [15,16,17]. In the Spanish cohort, GLE/PIB showed a higher prevalence of DDIs (17.8%), which was followed distantly by SOF/VEL (2.8%) [15]. A possible explanation relies on the different mechanisms of action of GLE/PIB, which led to a number of important potential drug interactions [19]. In our study, the two cohorts differ in the potential of DDIs, especially in the case of contraindicated co-treatments, which were less administered during SOF/VEL exposure (0.4%) than GLE/PIB (3.2%). The same tendency was reported in the literature; Schulte et al. [6] showed that contraindications due to DDIs interested 2% of SOF/VEL and 4% of GLE/PIB patients, while Sicras et al. [15] observed that SOF/VEL presented a lower percentage of medication contraindicated compared to GLE/PIB (1.7% vs. 8.3%). There has been reported a greater number of contraindications with cardiovascular co-treatments associated to GLE/PIB vs. SOF/VEL in the Spanish cohort (12.8% GLE/PIB vs. 1.4%) [17]. These data are aligned with our results (2.7% GLE/PIB vs. few patients with SOF/VEL). Among GLE/PIB patients, contraindicated co-treatments are mainly represented by lipid-modifying drugs (statins). As for lipid modifying co-treatments with significant DDI, SOF/VEL patients were mostly treated with atorvastatin, who actually do not require dose adjustment of SOF/VEL, according to the EU label. For GLE/PIB, the significant CV DDIs (5.9%) involved blood pressure medication.

Regarding CNS drugs, antiepileptics drugs were contraindicated in both pDAAs with ≤5 cases. However, the most common medications with significant CNS DDIs were found in the GLE/PIB cohort (2%): antipsychotics (1.3%) represented by quetiapine (1.1%) and analgesics (0.7%) led by oxycodone (0.6%). Both CNS drugs were also frequently found in the HCV patients Spanish cohort [16].

On the other hand, the SOF/VEL regimen seemed to have more significant interactions that are mainly due to PPIs. Patients treated with SOF/VEL have a multi-morbid profile, thus requiring multiple treatments, so they were probably taking PPIs for gastroprotection reasons.

Negative consequences of drug interactions with DAA may include decreased concentrations resulting in loss of efficacy, or the contrary, increased levels associated to drug toxicity. Drugs used for HCV patients with cardiovascular disease, as statins, are substrates of various drug transporters and drug-metabolizing enzymes that are inhibited by specific DAAs, resulting in a clinically relevant increase in statin plasma concentrations and consequently potential safety issues [20]. In the same way, the use of some CNS as psychoactive agents during DAA therapy can increase the risk of DDIs. Many DAAs and psychoactive agents are extensively metabolized in the liver and have the ability to affect the activities of various enzymes (CYP450) and drug transporters. This makes DAAs as well as psychoactive agents possible victims (objects of DDIs) and perpetrators (causes of DDIs) of drug interactions, which could negatively affect treatment outcomes as a result of adverse effects (increased plasma concentrations) or treatment failure (decreased plasma concentrations) [21].

Conversely, PPIs, one of the most important alimentary tract and metabolism drugs prescribed in HCV patients, increase gastric pH and may affect DAAs bioavailability [7,14,15]. However, different studies provide reassurance that the co-administration of DAAs and PPI does not negatively affect the chance of viral eradication [14,22].

For DAAs with protease inhibitors, potential DDIs should be checked before recommending their use; NS5A protein inhibitors are potent and effective but have a low resistance barrier and variable toxicity profiles, while NS5B polymerase inhibitors have a high genetic barrier, and their metabolism generally does not depend on cytochrome P450 [16]. In general, regimens with the NS5B inhibitor sofosbuvir plus an HCV NS5A inhibitor, which do not affect CYP450, were relatively free of significant pharmacokinetic interactions, even in patients with moderate to severe liver impairment [7]. Actually, recent reviews reported that the administration of drugs concomitantly with SOF generally resulted in fewer DDIs than with protease inhibitor-based regimens [15,16,23]. Currently, the most widely used DAAs demonstrate a moderate DDI risk profile, which is significantly lower compared with first-generation protease inhibitors [24,25]. However, it has been recently shown that despite these advantages, the overall frequency of DDIs in the real-world analysis remained more or less stable over the treatment periods, with about 40% of HCV patients affected [6].

Within the study population, SOF/VEL requires less changes of contraindicated concomitant drugs before starting pDAA treatment due to potential DDIs. Dose modification, switch, or interruption rates are different among pDAAs, suggesting a different perception regarding the potential severity on DDIs. The adjustment of co-medications observed in both cohorts suggests that DDIs are assessed when DAAs are prescribed. However, there is still room for improvement, since a number of patients were still receiving contraindicated co-treatment, although most of them had a dose decrease. DDI risk in DAA-treated patients is linked to co-medication, but uncertainties remain over safety or clinical impact. The clinical relevance of DDI suggests that awareness in the administration of co-medications should be increased. Attention should be given to widespread major DDIs and their potential adverse outcomes. Moreover, specialists should not only be aware of the principles of dose adjustment in patients with hepatitis but also the clinically significant DDIs of the drugs used to treat hepatitis and comorbid illnesses in this population. Similarly, physicians will be able to choose the appropriate DAA regimen with the least number of DDIs for HCV patients. In this direction, real-world study assessing the potential DDI among HCV patients on pDAA treatment could be a valid tool for health professionals in their clinical practice to optimize the appropriateness of prescriptions.

We acknowledge some limitations of the study. Our cohort of patients reflected real clinical practice, and the results must be interpreted taking into account the limitations related to the observational nature of the study, which was based on data collected from administrative databases. The first limitation was the lack of clinical information related to the severity of the pathology in terms of HCV stages and other potential confounders that could have influenced our results. Secondly, data regarding pharmacological treatments were retrieved from pharmaceutical databases; therefore, the actual use of drugs was not available. Moreover, pharmacological databases do not provide information on drugs prescribed during hospitalizations. Laboratory tests or HCV prescription were used as proxy to estimate time to diagnosis, which hence could be longer than the time observed. Ultimately, the co-treatments and the DDIs could be underestimated, since administrative databases contain data on healthcare resources reimbursed by the Italian National Health Service and out-of-pocket therapies cannot be traced.

## 5. Conclusions

This real-world study showed that HCV patients present a clinical and demographic profile that could potentially expose them to DDI when initiating pDAA therapy. In our study population, the most commonly prescribed therapeutic groups with contraindicated DDIs are those related to the cardiovascular and nervous system. Our findings highlight that SOF/VEL is used preferably in older patients, with higher rates of comorbidities and comedications, which are mainly related to cardiovascular and nervous system comorbidities. Despite this, a lower proportion of contraindicated drugs was registered compared to GLE/PIB. The pharmacoutilization of co-treatments revealed that although a DDI assessment was observed through rates of discontinuations or dose decrease, there are areas of improvement in the management of co-treatments during pDAA exposure, especially for contraindicated ones, for which efforts are needed to avoid uncertain clinical consequences due to DDIs.

## Figures and Tables

**Table 1:** Demographic and clinical characteristics at baseline.

Characteristics	SOF/VEL	GLE/PIB	*p*-Value
N	1562	1619	
Age (mean, SD)	58.4 (15.8)	53.1 (15.6)	<0.001
<40	160 (10.2)	283 (17.5)	<0.001
40–49	252 (16.1)	386 (23.8)	
50–59	497 (31.8)	488 (30.1)	
60–69	199 (12.7)	171 (10.6)	
70–79	285 (18.2)	196 (12.1)	
80+	169 (10.8)	95 (5.9)	
Male (n, %)	911 (58.3)	963 (59.5)	0.530
Charlson Index (mean, SD)	0.9 (1.3)	0.6 (1.0)	<0.001
Cirrhosis (n, %)	238 (15.2)	117 (7.2)	<0.001
HCC (n, %)	29 (1.9)	5 (0.3)	<0.001
Liver transplant (n, %)	6 (0.4)	0 (0.0)	0.037
Previous HCV treatment (n, %)	79 (5.1)	46 (2.8)	0.002
CV comorbidities	909 (58.2)	685 (42.3)	<0.001
CNS comorbidities	521 (33.4)	457 (28.2)	0.002

Abbreviation: CNS, central nervous system; CV, cardiovascular; GLE/PIB: glecaprevir/pibrentasvir; HCC hepatocellular carcinoma; HCV: hepatitis C virus; SOF/VEL: sofosbuvir/velpatasvir.

**Table 2:** Persistence and changes in concomitant contraindicated CV and CNS drugs during DAA therapy.

Class	Pts N	Persistence N	Adherence N	Dose Decrease N	Discontinuation N	Resumption N
SOF/VEL cohort						
CV	16	0	0	0	16	NI *
CNS	17	NI *	NI *	NI *	14	NI *
GLE/PIB cohort						
CV	136	42	41	40	94	41
CNS	15	4	4	4	11	0

Abbreviation: CNS, central nervous system; CV, cardiovascular; SOF/VEL: sofosbuvir/velpatasvir; GLE/PIB: glecaprevir/pibrentasvir. * Following the “Opinion 05/2014 on Anonymization Techniques” drafted by the “European Commission Article 29 Working Party”, the analyses involving less than three patients were not reported, as they were potentially traceable to single individuals. Therefore, results referred to ≤3 patients were reported as NI (not issuable).

## Data Availability

All data used for the current study are available upon reasonable request next to CliCon s.r.l. which is the body entitled of data treatment and analysis by Local Health Units.

## Supplementary Materials

The following are available online at https://www.mdpi.com/article/10.3390/ijerph18137144/s1, Figure S1: Comparison (#pts) between DAAs based on the risk of DDIs with nervous system and cardiovascular most common concomitant drugs.
