# Supplementary material for: Drug–Drug Interactions in Italian Patients with Chronic Hepatitis C Treated with Pangenotypic Direct Acting Agents: Insights from a Real-World Study
Source: Int J Environ Res Public Health. 2021 Jul 3;18(13):7144. doi: 10.3390/ijerph18137144 (PMC8296917; doi:10.3390/ijerph18137144)
Supplement: Supplementary file 1 [file ijerph-18-07144-s001.zip › ijerph-1235390-supplementary.pdf]

Supplementary material

Supplementary Figure S1. Comparison (#pts) between DAAs based on the risk of DDIs with CNS and CV most common concomitant drugs.

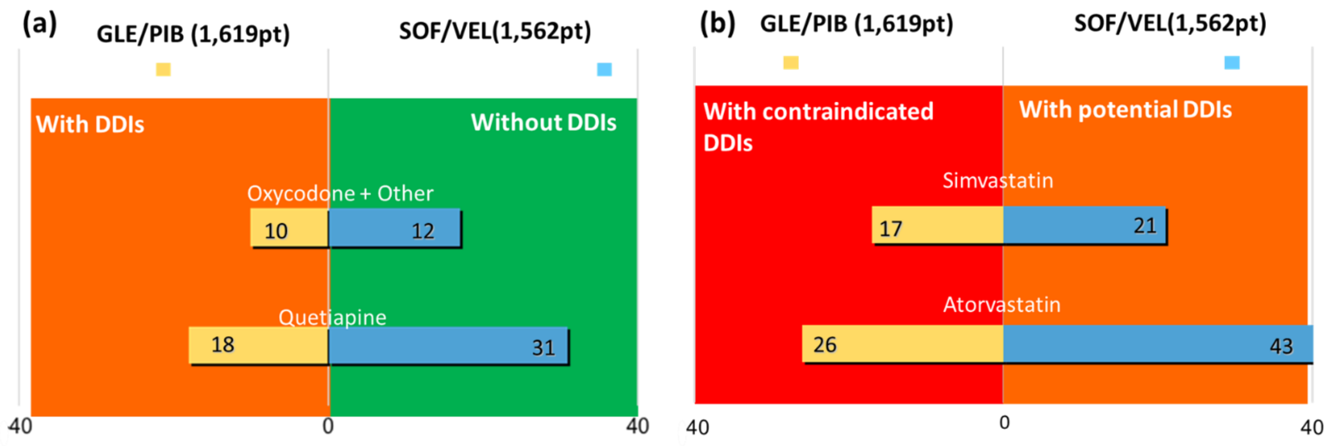

Abbreviations: DDI, drug-drug interaction; GLE/PIB: glecaprevir/pibrentasvir; SOF/VEL: sofosbuvir/velpatasvir.
